# Synergistic antitumor activity of baicalein combined with almonertinib in almonertinib-resistant non-small cell lung cancer cells through the reactive oxygen species-mediated PI3K/Akt pathway

**DOI:** 10.3389/fphar.2024.1405521

**Published:** 2024-07-31

**Authors:** Teng Chen, Pei Zhang, Xiao-Fan Cong, Yuan-Yuan Wang, Shuo Li, Hao Wang, Su-Rong Zhao, Xiao-Jin Sun

**Affiliations:** ^1^ School of Pharmacy, Bengbu Medical University, Bengbu, China; ^2^ Anhui Engineering Technology Research Center of Biochemical Pharmaceuticals, Bengbu, China

**Keywords:** baicalein, almonertinib, resistance, non-small cell lung cancer, apoptosis, reactive oxygen species, PI3K/Akt signaling pathway

## Abstract

**Introduction:**

Almonertinib is an important third-generation epidermal growth factor receptor tyrosine kinase inhibitor (EGFR-TKI) exhibiting high selectivity to EGFR-sensitizing and T790M-resistant mutations. Almonertinib resistance is a major obstacle in clinical use. Baicalein possesses antitumor properties, but its mechanism of antitumor action against almonertinib-resistant non-small cell lung cancer (NSCLC) remains unelucidated.

**Methods:**

CCK-8 assay was used to examine the survival rate of H1975/AR and HCC827/AR cells following treatment for 24 h with different concentrations of baicalein, almonertinib or their combination. The changes in colony formation ability, apoptosis, and intracellular reactive oxygen species (ROS) levels of the treated cells were analyzed using colony formation assay and flow cytometry. Western blotting was performed to detect the changes in protein expressions in the cells. The effects of pre-treatment with NAC on proliferation, apoptosis, and PI3K/Akt signaling pathway were observed in baicalein- and/or almonertinib-treated cells. A nude mouse model bearing subcutaneous HCC827/AR cell xenograft were treated with baicalein (20 mg/kg) or almonertinib (15 mg/kg), and the tumor volume and body mass changes was measured.

**Results:**

Both baicalein and almonertinib represses the viability of HCC827/AR and H1975/AR cells in a concentration-dependent manner. Compared with baicalein or almonertinib alone, the combined application of the two drugs dramatically attenuates cell proliferation; triggers apoptosis; causes cleavage of Caspase-3, PARP, and Caspase-9; downregulates the protein expressions of p-PI3K and p-Akt; and significantly inhibits tumor growth in nude mice. Furthermore, baicalein combined with almonertinib results in massive accumulation of reactive oxygen species (ROS) and preincubation with N-acetyl-L-cysteine (ROS remover) prevents proliferation as well as inhibits apoptosis induction, with partial recovery of the decline of p-PI3K and p-Akt.

**Discussion:**

The combination of baicalein and almonertinib can improve the antitumor activity in almonertinib-resistant NSCLC through the ROS-mediated PI3K/Akt pathway.

## Graphical Abstract

**Figure:**
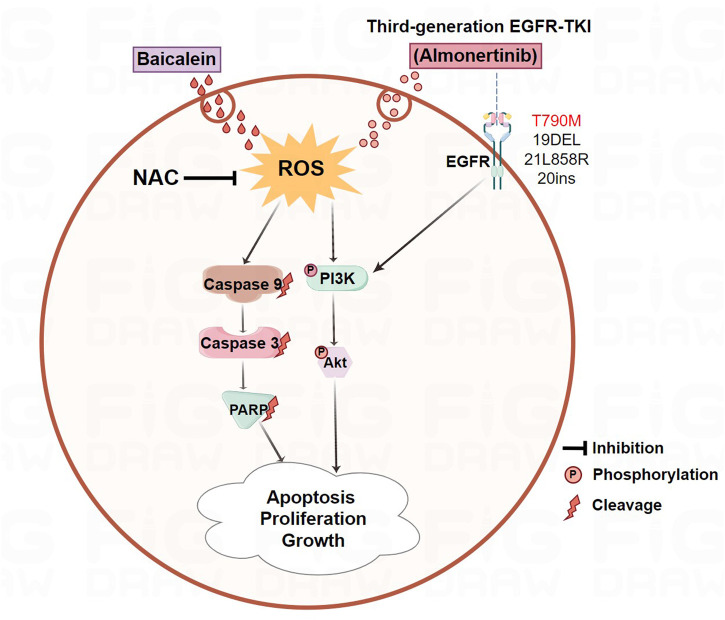
The combination of baicalein and almonertinib improved antitumor activity in almonertinib-resistant NSCLC through ROS-mediated PI3K/Akt pathway.

## 1 Introduction

Lung cancer is one of the main reasons for tumor-related deaths around the world, and its numbers continue to increase (Bray et al., 2018). Non-small cell lung cancer (NSCLC) is a common type of lung carcinoma that accounts for 85% of all incidences of lung cancers (Molina et al., 2008). Most of these patients were at advanced stages, with 5-year survival rate of less than 5% when diagnosed (Chinese Society of Clinical Oncology et al., 2019; Ye et al., 2021). Traditionally, chemotherapy and surgical resections are the commonly employed treatments for cancer, but these have poor efficacies; targeted therapy has therefore become increasingly important for treating NSCLC. Currently, epidermal growth factor receptor (EGFR) is a crucial molecule for targeted therapy (Naylor et al., 2016); EGFR tyrosine kinase inhibitors (EGFR-TKIs) have been extensively adopted in EGFR-mutated NSCLC patients. To date, three generations of EGFR-TKIs have been applied in clinics, and the third-generation EGFR-TKI is the most widely used therapy, which can irreversibly bind to the mutant EGFR (T790M, L858R, and exon 19 deletion) to overcome acquired resistance of the first- and second-generation EGFR-TKIs (Hsu et al., 2018; Andrews Wright and Goss, 2019; Park et al., 2021; Passaro et al., 2021). Almonertinib is the first innovative third-generation EGFR-TKI in China, which shows stronger inhibitory activities against both T790M mutations and EGFR sensitization while having limited effects on wild-type (WT) EGFR (Cross et al., 2014; Mok et al., 2017; Reungwetwattana et al., 2018; Soria et al., 2018), and it has provided a new option for treating NSCLC patients in China. However, the acquired resistance to almonertinib is considered the main barrier for the treatment of NSCLC, so innovative and effective therapies are increasingly being required to mitigate such acquired resistance to almonertinib.

Recently, many traditional Chinese medicines and their active constituents have been verified to exhibit synergistic effects with chemotherapeutic drugs. Baicalein, also known as 5,6,7-trihydroxyflavone, is a polyhydroxyflavonoid compound extracted and isolated from Radix Scutellariae that has been found to exert multiple effects, such as antitumor, anti-inflammatory, and antioxidant activities (Li et al., 2000; Liu et al., 2003; Burnett et al., 2007; Peng-Fei et al., 2013; Chen et al., 2021). It has been particularly shown to play essential roles in antitumor treatments, such as repressing cell growth and metastasis, suppressing cell invasion and migration, and mediating apoptosis and autophagy (Wang et al., 2014; Guo et al., 2015; Yan et al., 2015; Bie et al., 2017). Previous studies have shown that baicalein has inhibitory effects on lung cancer cell proliferation and metastasis (You et al., 2018; Gu et al., 2022). Nevertheless, the mechanism behind its antitumor actions against almonertinib-resistant NSCLC remains unascertained. In this study, the effects and related actions of the mechanism of baicalein combined with almonertinib on almonertinib-resistant NSCLC were evaluated through network pharmacology, *in vitro*, and *in vivo* assays.

## 2 Materials and methods

### 2.1 Reagents and antibodies

Almonertinib was provided by Jiangsu Hausen Pharmaceutical Co., Ltd. Baicalein (CAS:491-67-8, MCE) was obtained from MedChemExpress (Shanghai, China). Cell counting kit-8 (CCK-8) was procured from APExBIO (Houston, USA). The annexin V-FITC/PI staining kit was purchased from Bestbio (Shanghai, China). The EdU, BCA, and reactive oxygen species (ROS) kits were supplied by Beyotime (Shanghai, China). The antibody against PARP (#T40050) was purchased from Abmart. The antibodies of GAPDH (60004-1-Ig) and β-tubulin (10094-1-AP) were purchased from Proteintech. The antibody against PI3K (BS6052) was obtained from Bioward. The antibodies of p-PI3K (#4249), Akt (#4691), p-Akt (#4060), Caspase-3 (#14220), Caspase-9 (#9502), cleaved Caspase-3 (#9661), and cleaved Caspase-9 (#7237) were supplied by Cell Signaling Technology.

### 2.2 Cell culture and establishment of drug-resistant cell lines

H1975 and HCC827 cells in the logarithmic growth phase (with cell fusion of 70%–80%) were collected and exposed to 2 μM of almonertinib-containing medium initially. After 24 h, the medium was discarded and cells were rinsed twice with phosphate-buffered saline (PBS) before switching to a normal medium without almonertinib. Once the cells resumed their growth, they were subcultured. When the cells achieved a certain level of fusion, 2 μM of almonertinib was reapplied. Once the cells stably grew at this drug concentration, the concentration of almonertinib was gradually increased for continued cultivation. Cells were typically cultured for 1–2 weeks at each concentration level. As the drug concentrations were continuously increased, constant monitoring of the cell sensitivity was required to ensure that the cells retained their drug resistance. The drug induction process lasted for 6 months. The H1975 and HCC827 parent cells and their almonertinib-resistant equivalents grew in RPMI-1640 medium with 1% penicillin/streptomycin solution and 10% fetal bovine serum (FBS) in a constant-temperature incubator at 37°C and 5% CO_2_.

### 2.3 Cell viability analysis

The cells were seeded in 96-well plates at 5,000 cells per well. Twenty-four hours later, the cells were subjected to 0, 1, 2, 4, 8, 16, and 32 μM of almonertinib or 0, 20, 40, 60, 80, and 100 μM of baicalein; a blank group and a control group were also set up simultaneously. After 24 h of cultivation, the cell media were discarded carefully and the CCK-8 kit was added to the wells; then, the cells were incubated in the dark at 37°C for 30 min. The optical density (OD) value (at 450 nm) was obtained, and the cell viability was analyzed according to the OD value as described previously (Lei et al., 2023). The half maximal inhibitory concentration (IC_50_) value was acquired based on the OD value using the GraphPad Prism 8 software.

### 2.4 Synergy analysis

The concentration range of almonertinib or baicalein was premeasured via the IC_50_ value of each agent, and the cell viability was determined based on the concentration of the two drugs (almonertinib: 1, 2, 4, 8, 16, and 32 μM; baicalein: 40, 60, 80, 100, and 120 μM). Twenty-four hours later, the cell viabilities were analyzed with the CCK-8 kit. The online software SynergyFinder (https://synergyfinder.fimm.fi) was used to obtain the zero potency (ZIP) based on the OD value. Synergism was considered when the ZIP synergy scores exceeded 0, and strong synergistic effects were assumed when the scores exceeded 10.

### 2.5 Colony formation assay

Each well of a 6-well plate was inoculated with 1×10^3^ cells. After 3 days in the incubator, fresh media containing almonertinib or/and baicalein were added for another 10–12 days. When the colonies were uniformly formed, the cells were processed as mentioned previously, and the counts of the cell colonies were analyzed using ImageJ software.

### 2.6 EdU assay

Each well of a 24-well plate was seeded with 5×10^4^ cells. The cells were processed according to the manufacturer’s instructions and examined with a fluorescence microscope. The ImageJ software was applied to evaluate the percentage of EdU-positive cells.

### 2.7 Apoptosis assay

The Annexin V-FITC/PI staining was used to observe the apoptosis. Each well of a 6-well plate was inoculated with 2×10^5^ cells and supplied with almonertinib (8 μM) or/and baicalein (80 μM or 60 μM) for 24 h. The cells were processed according to the manufacturer’s instructions and detected using a flow cytometer, then subsequent determination with FlowJo software.

### 2.8 Western blotting

The cells were placed in culture plates with appropriate cell densities. After incubation with almonertinib (8 μM) or/and baicalein (80 μM or 60 μM) for 24 h, the cells were lysed on ice in RIPA buffer. After measuring the protein concentration via the bicinchoninic acid protein assay kit, SDS-PAGE was performed to separate the equivalent proteins from all groups; the proteins were moved to membranes that were then blocked with 5% skim milk, treated with primary antibodies, reacted with secondary antibodies, visualized using ECL chemiluminescent reagent, photographed with a gel imaging instrument, and quantified for the bands using ImageJ software. GAPDH or β-tubulin was used as the loading reference.

### 2.9 Intracellular ROS level detection

The fluorescent probe 2′,7′-dichlorofluorescin diacetate (DCFH-DA) was employed to evaluate the intracellular ROS level. The cells grown in 6-well plates and processed with almonertinib (8 μM) or/and baicalein (80 μM or 60 μM) for 24 h were subsequently treated with DCFH-DA (10 μM) for 20 min, and the level of DCF fluorescence of the cells was determined via flow cytometry. To prevent ROS production, a ROS remover like N-acetyl-L-cysteine (NAC) (2 mM) was applied for 1 h before treatment with almonertinib or/and baicalein to the cells. The results were then analyzed with FlowJo software.

### 2.10 Xenograft mouse model

Five-week-old female BALB/c nude mice of 18–20 g body weight were supplied by Hangzhou Ziyuan Experimental Animal Technology Co., Ltd. (experimental animal quality certificate of license No. SCXK2019-0004). All mice were fed under specific pathogen-free conditions, and food and water were made freely available in the cage. The temperature and humidity were maintained at (25 ± 2)°C and (50 ± 5)%, respectively. The animal study was approved by the Ethics Committee of Bengbu Medical University (approval no. 2022A170). A total of 5×10^6^ HCC827/AR cells were inoculated into each of the nude mice subcutaneously. Seven days later, each animal was assigned to one of four groups at random: control group, baicalein (20 mg/kg) group, almonertinib (15 mg/kg) group, and baicalein (20 mg/kg) combined with almonertinib (15 mg/kg) group. The body weight of each mouse and its tumor size were determined once every 3 days, and the tumor volume was obtained as follows: V = length × width × width/2. Twenty-four days later, tumor samples were collected from the mice for the TUNEL assay and immunohistochemistry (IHC) analysis; the liver, kidney, heart, spleen, and lung of each mouse were obtained for hematoxylin and eosin (HE) staining; blood was also drawn for detecting the levels of alanine aminotransferase (ALT), aspartate transaminase (AST), blood urea nitrogen (BUN), and creatinine (Cr).

### 2.11 TUNEL staining assay

The tumor slides were processed with protease K and reacted with Alexa Fluor 488-12-dUTP in the dark. The stained slides were dyed with DAPI solution and subsequently observed under a microscope.

### 2.12 HE staining

The tissue specimens were made transparent using xylene before cooling in melted paraffin and solidifying to wax blocks that were cut into slices of thickness 4 μm. The slices were subject to HE staining as reported previously (Ma et al., 2024) and observed and photographed under a microscope.

### 2.13 IHC analysis

The tumor sections were subject to 3% hydrogen peroxide and blocked with serum; subsequently, they were probed overnight with anti-Ki67, anti-cleaved Caspase-3, anti-p-PI3K, and anti-p-Akt antibodies before being reacted with secondary antibodies, followed by DAB staining and examination under a confocal microscope.

### 2.14 Network pharmacology

#### 2.14.1 Screening of potential targets

All targets of baicalein were acquired from the Swiss Target Prediction Database (http://www.swisstargetprediction.ch/) and SuperPred Database (https://prediction.charite.de/). These protein targets were uniformly normalized in UniProt (http://www.uni-prot.org/) after deduplication. The lung cancer-related targets were obtained from GeneCards (http://www.GeneCards.org/) and OMIM (https://OMIM.org/). The common targets between baicalein and lung cancer were identified using Cytoscape 3.7.2 software.

#### 2.14.2 Protein–protein interaction (PPI) network establishment

In an effort to further investigate the PPIs between baicalein and lung cancer, the PPI network was generated by importing the common targets into the String database (https://string-db.org/) and analyzed as mentioned previously (Guo et al., 2021). The results were stored in TSV format and input to Cytoscape 3.7.2 software.

#### 2.14.3 Gene ontology (GO) and Kyoto encyclopedia of genes and genomes (KEGG) pathway enrichment analyses

The GO and KEGG pathway analyses were carried out to analyze the common protein targets between lung cancer and baicalein using the cluster Profiler package. The species was set as “Homosapiens”, with *p* < 0.05 being used as the criterion for enrichment screening, and the top-10 genes were selected to generate the bar and bubble charts with the Rstudio package “ggplot2”.

### 2.15 Statistical analysis

The data were evaluated and plotted with GraphPad Prism 8 and SPSS 27 software; the numerical data were exhibited as mean ± SD, and the one-way ANOVA and LSD t-test were carried out to observe the differences among the groups. The value of *p* < 0.05 was used to indicate differences with statistical significance.

## 3 Results

### 3.1 Establishment of almonertinib-resistant HCC827/AR and H1975/AR cells

**FIGURE 1:**
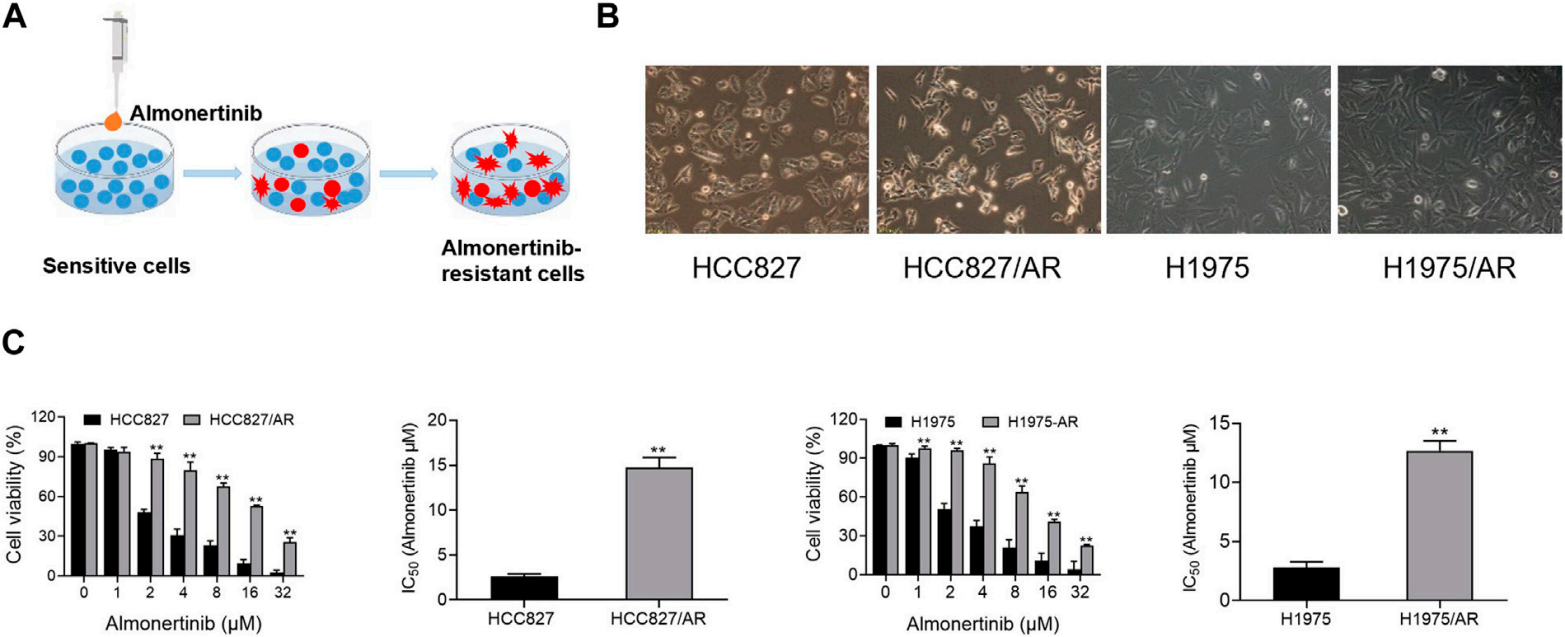
Construction and validation of the almonertinib-resistant HCC827/AR and H1975/AR cells. **(A)** Schematic of constructing the almonertinib-resistant H1975/AR and HCC827/AR cells. **(B)** Morphologies of the H1975 and HCC827 cells and their almonertinib-resistant counterparts observed under a microscope. **(C)** Cells were treated with the indicated concentrations of almonertinib for 24 h; the cell viabilities were determined via the CCK-8 assay, and the IC_50_ values were calculated.

The HCC827/AR and H1975/AR cells were derived from the HCC827 and H1975 cells (Wuhan Punosai Technology Co., Ltd.), respectively, by supplying almonertinib at increasing concentrations for about 6 months (Figure 1A). We found that both the HCC827 and H1975 parent cells, as well as the almonertinib-resistant cells, adhered to the walls. However, the HCC827/AR and H1975/AR cells were larger than their parent cells and displayed cluster-like growths, with some cells having elongated pseudopodia and irregular edges (Figure 1B). Moreover, CCK-8 assay was used to detect the resistances of the HCC827/AR and H1975/AR cells to almonertinib. The findings indicated that almonertinib dramatically decreased the survival rates of the HCC827 and H1975 cells and that high concentrations of almonertinib inhibited the survival rates of the HCC827/AR and H1975/AR cells. The IC_50_ values of the HCC827 and HCC827/AR cells to almonertinib were 2.59 ± 0.30 μM and 14.9 ± 1.38 μM, respectively, and the resistance index (RI) was 5.78 ± 0.65; the IC_50_ values of the H1975 and H1975/AR cells to almonertinib were 2.79 ± 0.49 μM and 12.67 ± 0.83 μM, respectively, and the RI was 4.63 ± 0.81 (Figure 1C). These findings implied that the drug-resistant cells were established successfully.

### 3.2 Baicalein and almonertinib inhibit proliferation of HCC827/AR and H1975/AR cells

**FIGURE 2:**
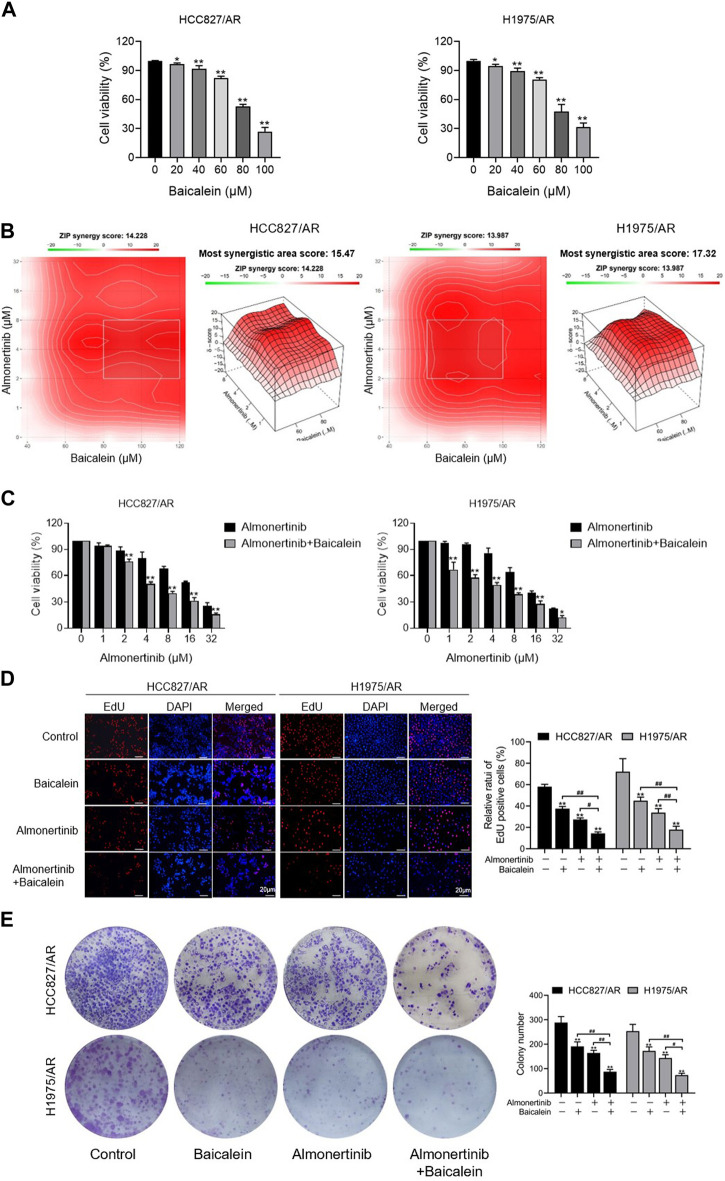
Effects of baicalein combined with almonertinib on the proliferations of the HCC827/AR and H1975/AR cells. **(A)** The HCC827/AR and H1975/AR cells were subjected to the indicated concentrations of baicalein; 24 h later, the cell viabilities were measured via the CCK-8 assay. The IC_50_ values of baicalein in the H1975/AR and HC827/AR cells were 81.01 ± 5.26 μM and 81.59 ± 1.37 μM, respectively. **(B)** Heatmaps of the drug combination responses. The cells were treated with the indicated concentrations of almonertinib and baicalein; 24 h later, the cell viabilities were acquired with the CCK-8 assay. ZIP synergy scores were obtained using the SynergyFinder software. **(C)** CCK-8, **(D)** EdU, and **(E)** colony formation tests were carried out to analyze the cell proliferations (n = 3, ^*^ *p* < 0.05, ^**^ *p* < 0.01, vs. control group).

In an effort to explore the impact of baicalein on cell viability, different concentrations of baicalein were used to process the HCC827/AR and H1975/AR cells, and the cell viabilities were dramatically restrained in a concentration-dependent manner (Figure 2A). To evaluate the effects of baicalein combined with almonertinib, baicalein (40, 60, 80, 100, and 120 μM) and almonertinib (0, 1, 2, 4, 8, 16, and 32 μM) were used to treat the cells; in the HCC827/AR and H1975/AR cells, cell proliferation was found to be remarkably suppressed. ZIP synergy scores were obtained with the SynergyFinder software, and the scores for the HCC827/AR and H1975/AR cells were 14.228 and 13.987, respectively (Figure 2B), indicating that almonertinib and baicalein had highly synergistic effects in suppressing cancer cell proliferation. Meanwhile, baicalein combined with almonertinib dramatically elevated the inhibition of cell viability (Figure 2C), and the EdU-positive cells (Figure 2D) were obviously reduced compared to the group treated with almonertinib or baicalein alone. Furthermore, the results of the colony formation assay suggested that baicalein and almonertinib in combination markedly declined the formation of colonies (Figure 2E). The above results indicated that baicalein and almonertinib in combination could obviously suppress the proliferation of almonertinib-resistant NSCLC cells.

### 3.3 Baicalein combined with almonertinib causes apoptosis in HCC827/AR and H1975/AR cells

**FIGURE 3:**
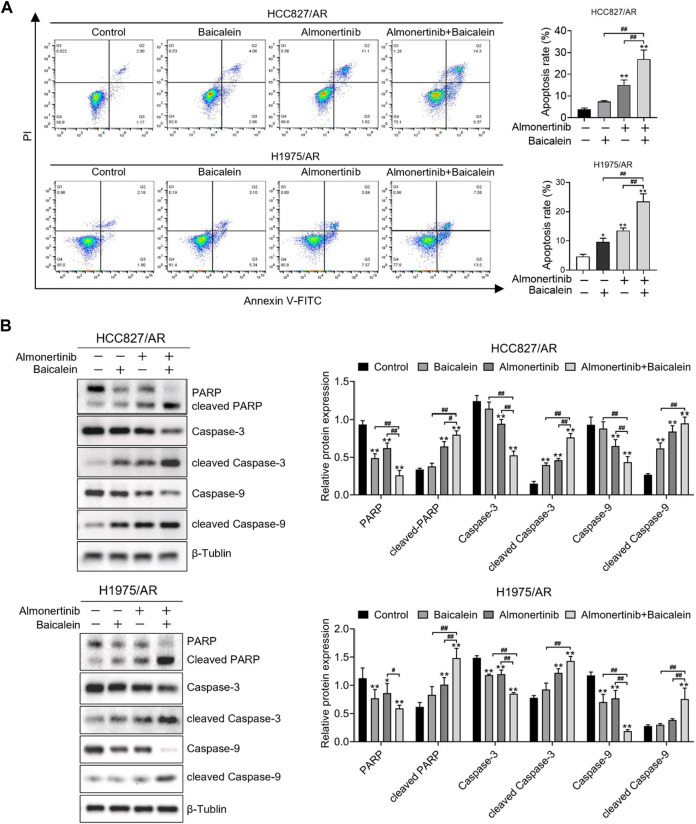
Baicalein combined with almonertinib promoted apoptosis in the HCC827/AR and H1975/AR cells. **(A)** The HCC827/AR cells were treated with baicalein (80 μM) and (or) almonertinib (8 μM) for 24 h, and the H1975/AR cells were treated with baicalein (60 μM) and (or) almonertinib (8 μM) for 24 h; then, the apoptosis rates were investigated via flow cytometry. **(B)** The expressions of the apoptosis-associated proteins (Caspase-3, PARP, and Caspase-9) were analyzed by Western blotting after treating the cells for 24 h, with β-tubulin as the internal reference. The gray values of the blots were acquired with ImageJ software (n = 3, ^*^ *p* < 0.05, ^**^ *p* < 0.01, and ^##^ *p* < 0.01, vs. control group).

We further explored the combined influences of baicalein and almonertinib on apoptosis in the HCC827/AR and H1975/AR cells, and the apoptosis rates of the cells were detected with the annexin-V/PI staining assay. Thus, the apoptosis rates of the cells processed with baicalein and almonertinib in combination were remarkably higher than that of the cells processed with a single drug (Figure 3A). Then, we conducted Western blotting to investigate the levels of several apoptosis-associated proteins and found that baicalein combined with almonertinib enhanced the cleaved Caspase-3, cleaved PARP, and cleaved Caspase-9 expressions in both H1975/AR and HCC827/AR cells, while decreasing the Caspase-3, PARP, and Caspase-9 expressions (Figure 3B). The above findings implied that baicalein and almonertinib triggered the activation of Caspase and then synergistically induce apoptosis.

### 3.4 Baicalein combined with almonertinib suppresses the growth of almonertinib-resistant NSCLC cells *in vivo*

**FIGURE 4:**
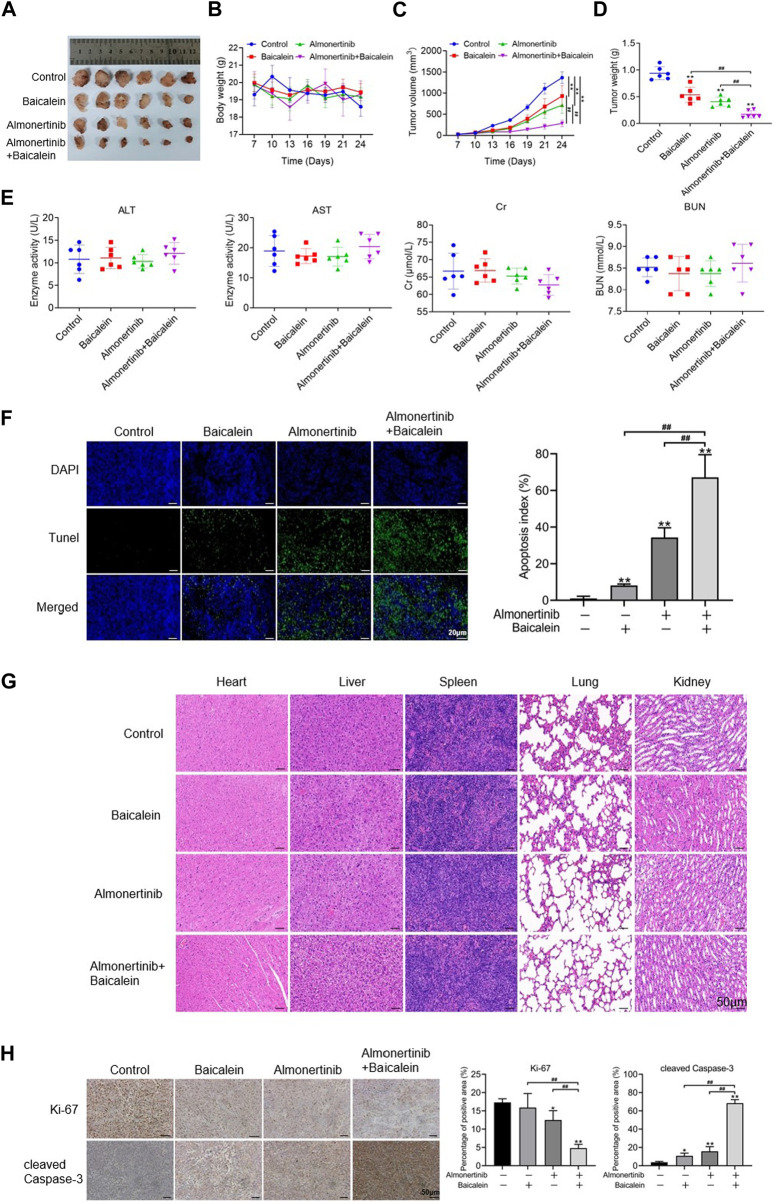
Effects of baicalein combined with almonertinib on the growths of HCC827/AR cell xenograft tumors. The HCC827/AR cells were subcutaneously administered at the neck and back of BALB/c nude mice (5 × 10^6^ cells in each mouse), followed by administration of different drugs for 24 days to investigate the antitumor activities of the drugs *in vivo*. **(A)** Tumor sizes, **(B)** tumor weights, **(C)** tumor volumes, and **(D)** body weight of the mice processed with baicalein (20 mg/kg) and almonertinib (15 mg/kg) alone or in combination. **(E)** ELISA was employed to determine changes in the indicators of renal function (BUN and Cr) and liver function (AST and ALT). **(F)** TUNEL assay was employed to analyze the apoptosis of tumor cells; scale bar = 20 μm. **(G)** HE staining graphs of the liver, kidney, heart, spleen, and lung; scale bar = 50 μm. **(H)** Representative immunohistochemistry graphs of Ki67 and cleaved Caspase-3; scale bar = 50 μm (n = 3, ^*^ *p* < 0.05, ^**^ *p* < 0.01, ^##^ *p* < 0.01).

The xenograft model experiments were performed with nude mice to ascertain the findings of the *in vitro* experiments. To determine the combined influences of baicalein and almonertinib on almonertinib-resistant NSCLC cells *in vivo*, a mouse xenograft model was established using the HCC827/AR cells. Compared to baicalein or almonertinib alone, the combination of two drugs significantly inhibited tumor growth (Figures 4A–C), even as no statistical differences were observed in the body weight of the nude mice (Figure 4D). To further observe the toxicity of liver and kidney functions in the nude mice, ELISA was applied to determine the liver and kidney function indicators, and the results suggested that both single and combined administrations of baicalein and almonertinib did not lead to kidney or liver toxicity (Figure 4E). Furthermore, the TUNEL staining results showed that baicalein combined with almonertinib dramatically elevated apoptosis in the tumor tissues in comparison with the groups receiving baicalein or almonertinib alone (Figure 4F). Otherwise, HE staining was employed to observe the structures and morphologies of the liver, spleen, heart, lung, and kidney, which displayed that these structures were intact without pathological changes (Figure 4G). Finally, we examined the level of proliferation indicator Ki67 and apoptosis indicator cleaved Caspase-3; in the tumor tissues, the findings implied that baicalein combined with almonertinib inhibited Ki67 expression and elevated the activation of Caspase-3 (Figure 4H), which were similar to the results acquired from the *in vitro* experiments. These findings showed that baicalein synergistically elevated the anticancer effects of almonertinib *in vivo* and that no significant hepatorenal toxicity was observed in any of the groups.

### 3.5 Baicalein combined with almonertinib enhances ROS levels in HCC827/AR and H1975/AR cells

**FIGURE 5:**
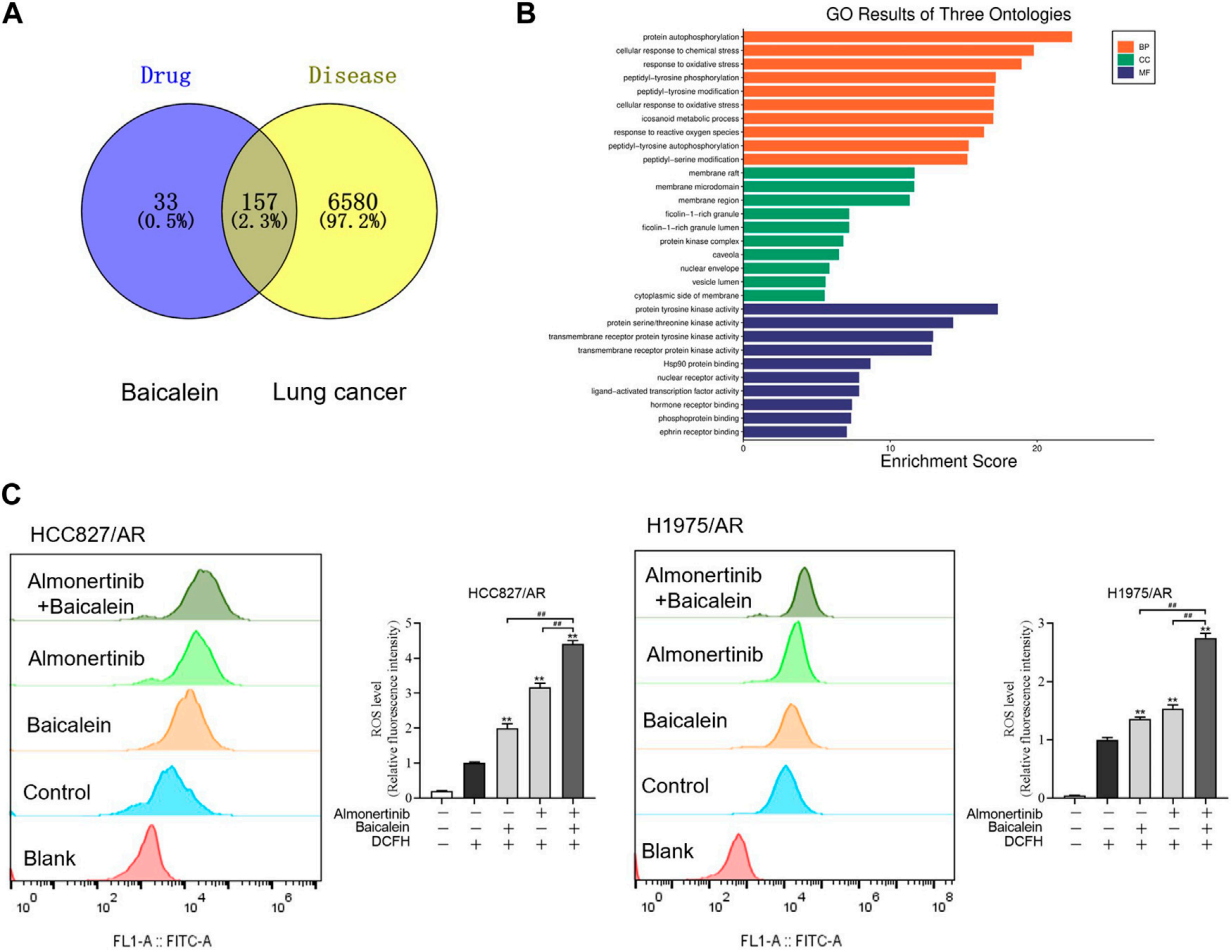
Baicalein combined with almonertinib increased intracellular ROS levels in the HCC827/AR and H1975/AR cells. **(A)** Venn diagram of the chemical targets of baicalein and lung cancer targets. **(B)** GO enrichment analysis of the common targets between baicalein and lung cancer. **(C)** Flow cytometry was carried out to measure the intracellular ROS levels in the HCC827/AR cells treated with baicalein (80 μM) and/or almonertinib (8 μM) as well as H1975/AR cells treated with baicalein (60 μM) and/or almonertinib (8 μM) for 24 h. ImageJ software was used to quantify the intracellular ROS levels (n = 3, ^**^ *p* < 0.01, ^##^ *p* < 0.01).

To explore the associations between baicalein and lung cancer, a total of 190 baicalein targets and 6,737 non-duplicate lung cancer targets were intersected to create a Venn diagram (Figure 5A), resulting in 157 intersecting target genes. A bar graph of the top 10 GO entries involving biological process (BP), cellular component (CC), and molecular function (MF) was created (Figure 5B). BP primarily covered reactions to oxidative stress, cellular responses to oxidative stress, and responses to ROS. Generally, ROS were produced and accumulated under oxidative stress, and the intracellular ROS affected various biological processes, including proliferation and apoptosis, by altering the equilibrium between cell survival and death processes. ROS generation was detected via DCFH-DA dyeing, followed by flow cytometry analysis of the ROS levels in the H1975/AR and HCC827/AR cells. As displayed in Figure 5C, the intracellular ROS levels in the combination group were dramatically higher than those in the single-drug groups.

### 3.6 ROS is implicated in the regulation of apoptosis and proliferation of HCC827/AR and H1975/AR cells upon combining baicalein and almonertinib

**FIGURE 6:**
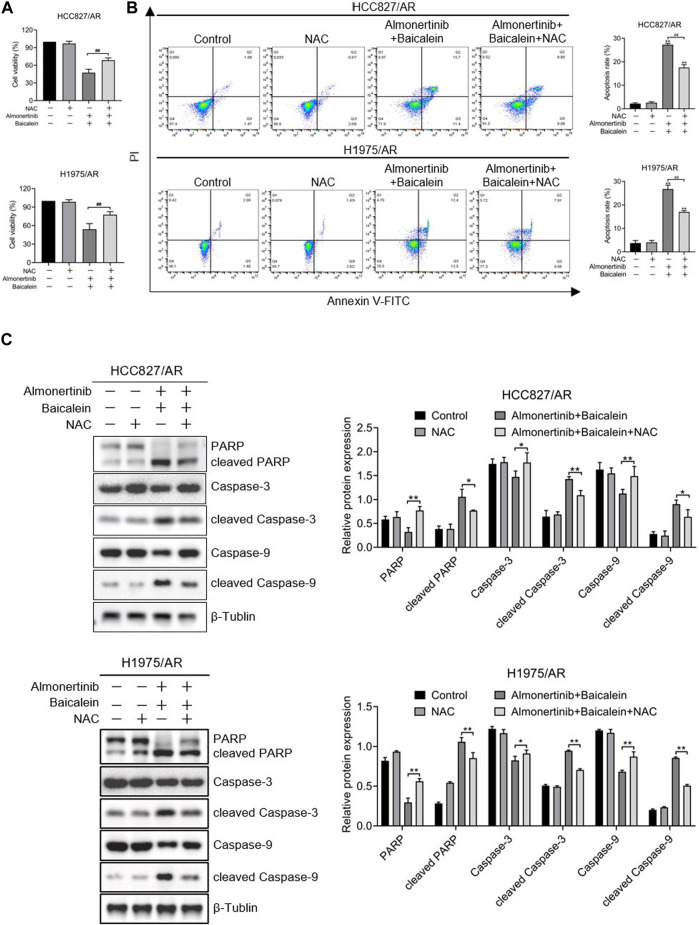
ROS played roles in affecting the apoptosis and proliferation of almonertinib-resistant NSCLC cells upon combined treatment with baicalein and almonertinib. The cells were preincubated with 2 mM of NAC for 1 h, and the HCC827/AR cells were treated with baicalein (80 μM) and almonertinib (8 μM), while the H1975/AR cells were treated with baicalein (60 μM) and almonertinib (8 μM) for 24 h. **(A)** The cell viabilities of the HCC827/AR and H1975/AR cells were examined via the CCK-8 assay. **(B)** Flow cytometry was carried out to detect apoptosis of the cells. **(C)** Western blotting was carried out to evaluate the expressions of PARP, Caspase-3, Caspase-9, cleaved PARP, cleaved Caspase-3, and cleaved Caspase-9 (n = 3, ^*^ *p* < 0.05, ^**^ *p* < 0.01, ^#^ *p* < 0.05, ^##^ *p* < 0.01).

To discuss the influences of ROS on proliferation inhibition and apoptosis induction in almonertinib-resistant NSCLC cells by baicalein combined with almonertinib, we hypothesized that ROS was a critical upstream mediator. The ROS inhibitor N-acetylcysteine (NAC) was used for cell pretreatment, and we found that NAC could partially reverse the combined influences of baicalein and almonertinib on proliferation inhibition (Figure 6A) and apoptosis induction (Figure 6B) in the HCC827/AR and H1975/AR cells. Meanwhile, the cleavages of PARP, Caspase-3, and Caspase-9 were also eliminated by NAC (Figure 6C). In summary, baicalein combined with almonertinib resulted in proliferation inhibition and apoptosis induction depending on ROS production.

### 3.7 Baicalein exhibits anticancer effects by modulating the PI3K/Akt pathway in almonertinib-resistant NSCLC cells

**FIGURE 7:**
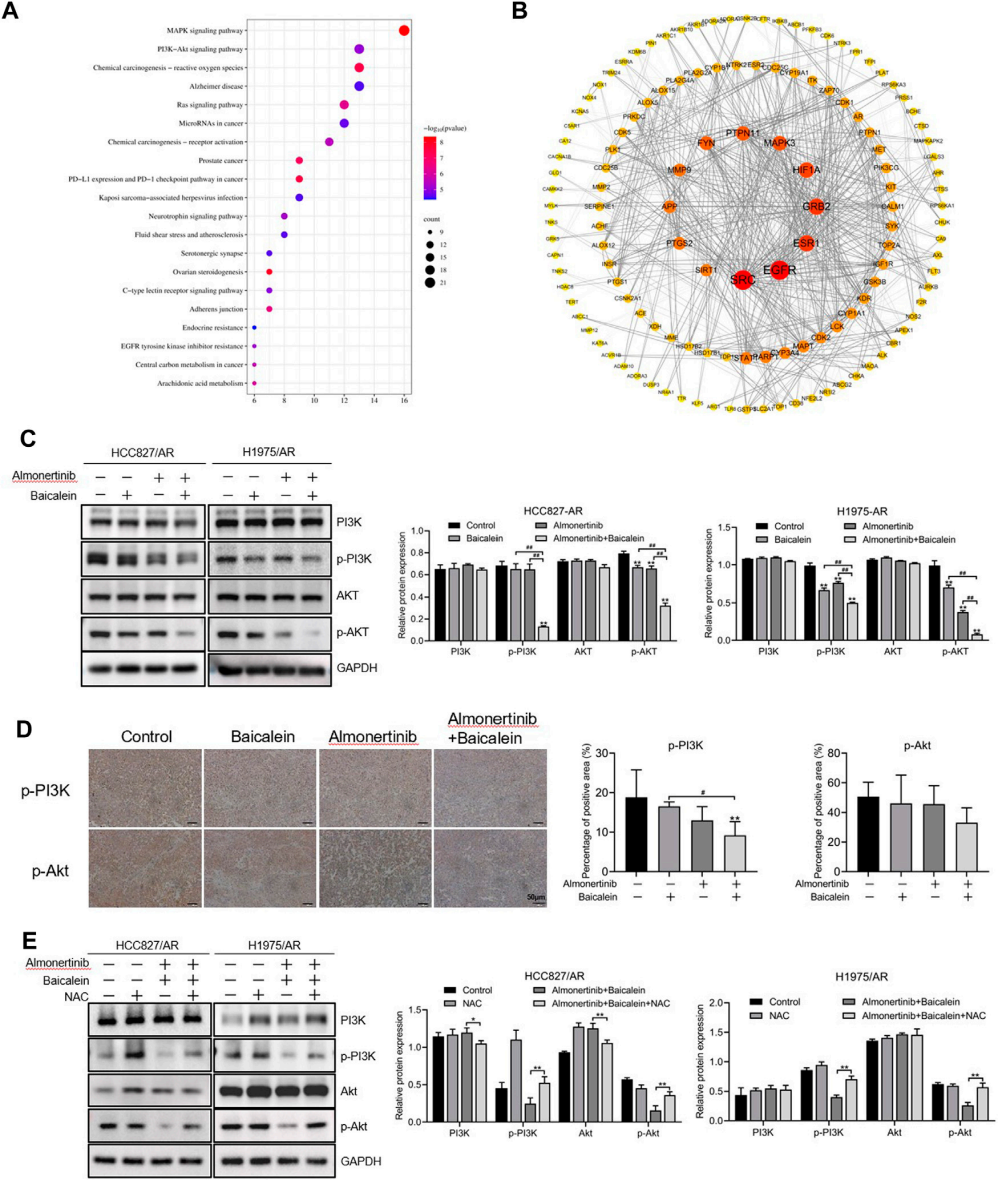
Effects of baicalein combined with almonertinib on the levels of PI3K/Akt-pathway-associated proteins in almonertinib-resistant NSCLC cells. **(A)** Top 20 enriched KEGG pathways of baicalein for the treatment of lung cancer. **(B)** PPI network from the common targets between baicalein and lung cancer. **(C)** Levels of the PI3K/Akt-pathway-associated proteins in the HCC827/AR and H1975/AR cells treated with baicalein and (or) almonertinib were evaluated by Western blotting using GAPDH as a loading reference. **(D)** Representative immunohistochemistry images of p-Akt and p-PI3K; scale bar = 50 μm. **(E)** Levels of the PI3K/Akt pathway-associated proteins in the HCC827/AR and H1975/AR cells after NAC pretreatment (n = 3, ^*^ *p* < 0.05, ^**^ *p* < 0.01, ^#^ *p* < 0.05, ^##^ *p* < 0.01).

The P13K/Akt pathway broadly exists in a wide range of cancers and exhibits essential modulating roles in cell survival, angiogenesis, and proliferation. As described in Section 3.5, we found 157 intersecting targets between baicalein and lung cancer. The KEGG and PPI pathways were employed to find the targets of baicalein for the treatment of lung carcinoma, and we found that the PI3K/Akt pathway played an essential role in treatment with baicalein in lung carcinoma (Figure 7A); the PPI network was created, as displayed in Figure 7B. Therefore, we speculate that the PI3K/Akt pathway serves as a vital pathway in the anticancer activity of baicalein combined with almonertinib in almonertinib-resistant NSCLC cells. To confirm this hypothesis, the levels of this pathway-associated protein in HCC827/AR and H1975/AR cells processed with baicalein and/or almonertinib were investigated. The findings revealed that the p-Akt and p-PI3K expressions were markedly downregulated in the groups treated with baicalein and/or almonertinib (Figure 7C). To further confirm the regulatory effects of baicalein combined with almonertinib on the PI3K/Akt pathway, IHC analysis was carried out to detect p-Akt and p-PI3K in the tumor tissues. The results showed that baicalein combined with almonertinib diminished the levels of p-Akt and p-PI3K, which were similar to the *in vitro* experimental results (Figure 7D). Furthermore, after pretreatment with NAC, the activation of PI3K and Akt was repressed, indicating that ROS participated in the PI3K/Akt pathway (Figure 7E). The above findings revealed that baicalein combined with almonertinib obviously suppressed activation of the PI3K/Akt pathway through ROS accumulation.

## 4 Discussion

Lung cancer is one of the most common carcinomas reported globally, with mutated EGFR being the usual driver of gene mutation. Almonertinib is an important third-generation tyrosine kinase inhibitor that selectively acts on EGFR mutations and is broadly applied in the treatment of NSCLC with EGFR mutations. The problem of resistance to almonertinib is still quite prominent, and multiple resistance mechanisms have been discovered in clinical practice, including secondary mutations and increased copy numbers of the EGFR gene. Therefore, solving the problem of resistance is very important to improve the efficacy of NSCLC treatment. It is also a good approach to establish *in vitro* resistant cancer cell lines to investigate the mechanisms of drug resistance (Watson et al., 2007). The almonertinib-resistant NSCLC HCC827/AR and H1975/AR cells were derived by gradually increasing almonertinib application concentrations to the parental HCC827 and H1975 cells in this study.

Numerous studies have found that baicalein has critical roles in NSCLC (Chandrashekar and Pandi, 2022; Gu et al., 2022); however, there are very few reports on its therapeutic effects and mechanisms against almonertinib-resistant NSCLC cells. We investigated the combined influences of baicalein and almonertinib on the proliferation and apoptosis of almonertinib-resistant NSCLC cells in this study, and the results suggested that both drugs could inhibit tumor proliferation, induce apoptosis, and had synergistic effects when used together.

Apoptosis is usually considered a hallmark of cancer (Pistritto et al., 2016), and it is a process wherein cell death is activated and modulated by the relevant genes to regulate normal tissue homeostasis (Chui et al., 2019). Cell death can be activated through both exogenous pathways (death receptor pathway, Caspase-8/10-dependent pathway) and endogenous pathways (cytochrome-C-induced mitochondrial pathway, Caspase-3/9-dependent pathway). Although these two pathways are mediated by different initiation factors, they ultimately require activation of the executing Caspase (Caspase-3) to induce changes in the downstream-related apoptotic factors and complete cell apoptosis, where Caspase-3 is the key to these relationships (Gaidt and Hornung, 2016). The cleavage of PARP is considered an indicator of Caspase-3 activation. Research has shown that baicalein mainly affects the levels of cytochrome C, Caspase-3, Bcl-2 family of proteins, Caspase-9, and others through various signaling pathways to affect endogenous apoptosis of the tumor cells (Gao et al., 2017; Wang et al., 2017). In this study, baicalein combined with almonertinib dramatically caused cleavages of Caspase-3, Caspase-9, and PARP compared to almonertinib or baicalein alone. Meanwhile, the expressions of PARP, Caspase-3, and Caspase-9 decreased, suggesting that apoptosis could be achieved through Caspase-9 and Caspase-3 cleavages inducing PARP cleavage, which confirmed that baicalein enhanced almonertinib-induced apoptosis in almonertinib-resistant NSCLC cells.

In an effort to study the regulatory mechanisms of the combined impacts of baicalein and almonertinib on apoptosis and proliferation, network pharmacology was employed to annotate the roles of the treatment targets of baicalein in lung cancer from the perspectives of BP, CC, and MF by GO enrichment analysis; this mainly involved oxidative stress and responses to the ROS, which were all related to the formation of ROS and could impact multiple biological processes such as tumor growth, proliferation, and apoptosis. ROS is an activator of multiple signaling pathways. Evidence has shown that certain ROS levels are beneficial for the proliferation and differentiation of tumor cells, but excessive ROS can lead to oxidative injury and inhibit the growth of tumor cells, making them more prone to oxidative stress and other reactions to initiate apoptosis (Cui et al., 2018; Srinivas et al., 2019). In this study, it was observed that the HCC827/AR and H1975/AR cells exhibited remarkably greater accumulation of ROS after processing with baicalein combined with almonertinib, which was partially reversed by pretreating the cells with the ROS scavenger NAC. Furthermore, proliferation inhibition and apoptosis induction by baicalein combined with almonertinib were eliminated by NAC. Meanwhile, the cleavages of Caspase-3, Caspase-9, and PARP were also inhibited. These data revealed that ROS had significant implications for the apoptosis of almonertinib-resistant NSCLC cells induced by baicalein and almonertinib. However, treatment with NAC did not completely reverse the toxic effects of baicalein combined with almonertinib on the NSCLC/AR cells. These results indicated that baicalein combined with almonertinib could cause apoptosis in almonertinib-resistant NSCLC cells through excessive ROS accumulation and that there was also an additional ROS-independent apoptotic pathway. In summary, baicalein combined with almonertinib induced apoptosis in the HCC827/AR and H1975/AR cells through both ROS-dependent and ROS-independent pathways.

The KEGG pathway enrichment analysis suggested that the common targets included four vital cancer-associated pathways, namely, the PI3K/Akt, MAPK, PD-1 checkpoint, and PD-L1 expression pathways, which might be vital for baicalein in the treatment of lung carcinomas. The PI3K/Akt pathway plays vital roles in regulating cell survival, and abnormal activation of this pathway is critically relevant to the incidence and progression of cancers as well as migration and chemoresistance of the cancer cells (Wei et al., 2020). PI3K is composed of a unique and conserved intracellular lipid kinase that can phosphorylate the 3’ hydroxy groups of phosphatidylinositol and phosphoinositol. Upon activation, PI3K can phosphorylate PIP2 on the plasma membrane, generating PIP3. This PIP3 binds to the pH domain at the Akt N-terminal, allowing the Akt to transfer from the cytoplasm to the cell membrane. Akt is a major downstream molecule of the PI3K/Akt pathway, which phosphorylates the downstream substrates upon activation and helps modulate multiple biological processes, such as apoptosis, cell proliferation, and cytoskeleton remodeling (Luo et al., 2003; Hemmings and Restuccia, 2012; Manning and Toker, 2017). We aimed to investigate the impacts of almonertinib and baicalein on the PI3K/Akt pathway in this study, and no significant differences were observed between the single-drug group and combination group; however, the p-PI3K and p-Akt levels were downregulated, which was more obviously inhibited in the combination group. This indicated that the combination of baicalein and almonertinib could effectively block the activation of Akt and PI3K in the PI3K/Akt pathway at the translation level, inhibiting the signal transmission through this pathway. In the above experiments, we found that baicalein combined with almonertinib could repress the proliferation of almonertinib-resistant NSCLC cells and cause apoptosis through intracellular ROS accumulation. To clarify the associations between the PI3K/Akt pathway and ROS, we applied NAC to alleviate the accumulation of ROS in the combination group, and the findings implied that NAC restored the inhibitory impacts on apoptosis in the combination group. Therefore, the inhibitory effects of baicalein combined with almonertinib on the PI3K/Akt pathway are mediated by the ROS.

These data were further confirmed *in vivo* through the subcutaneous tumor model in BALB/c nude mice. We found that almonertinib combined with baicalein could inhibit tumor growth evidently without adverse reactions on the other tissues and organs. Meanwhile, the combination of baicalein and almonertinib suppressed the levels of the p-Akt, p-PI3K, and Ki67 proteins and promoted cleaved Caspase-3 level *in vivo*, similar to the *in vitro* studies. Above all, baicalein combined with almonertinib has encouraging potential therapeutic effects on almonertinib-resistant NSCLC, suggesting its clinical therapeutic potential.

## 5 Conclusion

In this study, baicalein combined with almonertinib was shown to improve the antitumor activity in almonertinib-resistant NSCLC via the ROS-mediated PI3K/Akt pathway. These findings provide theoretical and experimental references for developing novel treatment strategies for almonertinib-resistant NSCLC.

## Data Availability

The datasets presented in this study can be found in online repositories. The names of the repositories and accession numbers can be found in the article/Supplementary Material.

## Abbreviations

EdU, 5-ethynyl-2′-deoxyuridine; NAC, N-acetyl-L-cysteine; PARP, poly ADP-ribose polymerase; PI, propidium iodide; PI3K, phosphatidylinositol 3 kinase; RI, resistance index; ROS, reactive oxygen species; FITC, fluorescein isothiocyanate; HE, hematoxylin and eosin.
